# Safety assessment of subtilisin QK in rats

**DOI:** 10.1186/s40360-021-00506-w

**Published:** 2021-06-26

**Authors:** Shuai Xiao, Dingbang Hu, Ya Gao, Yang Ai, Sang Luo, Song Chen, Ben Wang, Li Zhou, Yanshan Dong, Yefu Wang

**Affiliations:** 1 State Key Laboratory of Virology, Wuhan University School of Life Sciences, Wuhan, 430072 China; 2 Wuhan Zhenfu Pharmaceutical Co., Ltd., Wuhan, 430072 China; 3 Animal Biosafety Level III Laboratory, Wuhan University School of Medicine, Wuhan, 430072 China

**Keywords:** Subtilisin QK, Subchronic toxicity, Acute toxicity, Safety pharmacology, *Bacillus subtilis*

## Abstract

**Background:**

Subtilisin QK is a serine protease in the subtilisin family, and is fermented by *Bacillus subtilis QK02*. The fibrinolytic activity of subtilisin QK was measured by detecting low molecular weight degradation products using a spectrophotometric method developed by Japan Bio Science Laboratory Co., Ltd. Subtilisin QK powder can maintain its fibrinolytic activity for more than 24 months when it is stored at room temperature and protected from light. Our previous results showed that subtlisin QK directly degraded cross-linked fibrins in the fibrin plate assay and effectively inhibited thrombosis in the mouse thrombus model. The aim of this study was to determine the acute toxicity, potential subchronic toxicity, and safety pharmacology of subtilisin QK in Sprague–Dawley (SD) rats.

**Methods:**

In the acute toxicity study, a single oral dose of 100,000 FU/kg was administered to 10 female and 10 male SD rats. In the 28-day subchronic toxicity, 60 female and 60 male SD rats were randomly assigned to four experimental groups (daily oral dose of 0, 2500, 7500 and 25,000 FU/kg). In the safety pharmacology study, 20 female and 20 male SD rats were randomly assigned to four experimental groups (single oral dose of 0, 500, 1500 and 5000 FU/kg).

**Results:**

No death occurred and no adverse effects were observed in the acute toxicity study at a dose of 100,000 FU/kg. In the 28-day subchronic toxicity study, several hematological and blood biochemical parameters showed increases or decreases; however, due to the lack of a dose–response relationship, these differences were considered unrelated to treatment. In the safety pharmacology study, no adverse effects were observed on the central nervous of SD rats post-administration up to a dose of 5000 FU/kg subtilisin QK.

**Conclusion:**

The results showed that oral consumption of subtilisin QK is of low toxicological concern. No adverse effects were observed at doses of 2500, 7500, and 25,000 FU/kg in the 28-day subchronic toxicity, and the no-observed-adverse-effect level (NOAEL) of subtilisin QK was 25,000 FU/kg.

**Supplementary Information:**

The online version contains supplementary material available at 10.1186/s40360-021-00506-w.

## Background

Cardiovascular diseases (CVDs) are a major problem worldwide. The World Health Organization reported that 17.9 million people die from CVDs each year, representing 31% of all global deaths (https://www.who.int/en/news-room/fact-sheets/detail/cardiovascular-diseases-(cvds)). Thrombosis is the most important cause of CVDs; however, there is a lack of safe and effective oral thrombolytic drugs to prevent and treat those diseases. Bacterial subtilisins, a large class of microbial serine proteases, have significant effects on thrombolytic and anticoagulation with great exploitable potential as oral thrombolytic agents [1–3]. For example, nattokinase, which is produced by *Bacillus* subtilisin subsp. natto, is widely applied as functional food to prevent CVDs. In 1987, Japanese scholars first named “Nattokinase” (nattokinase), which has strong fibrinolytic activity [4]. Subsequently, nattokinase was found to efficiently dissolve thrombus in vitro and in vivo [5, 6]. In addition, it can also activate endogenous urokinase-type plasminogen activator and tissue plasmin activator (t-PA), reduce the levels of coagulation factors VII and VIII, and inhibit platelet aggregation [7–9]. Therefore, nattokinase can improve blood circulation and reduce the risk of thrombolysis. Volunteer experiments showed that a single oral dose of 2000 FU nattokinase had excellent thrombolysis and anticoagulation effects and no adverse effects [10, 11]. Nattokinase reportedly lowers blood pressure and reduces atherosclerosis in vivo [12, 13]. In another volunteer experiment, oral administration of 100 mg/day nattokinase for 8 weeks significantly reduced systolic and diastolic blood pressure [14]. Similar to nattokinase, subtilisin QK is also a serine protease produced by the fermentation of *Bacillus subtilis QK02*, and has better thrombolytic effects [15].

Subtilisin QK comprises 275 amino acids (molecular weight [MW] = 27.8 kDa), and is highly homologous to nattokinase (98.78%) [15]. It can degrade cross-linked fibrins and has strong thrombolytic capacity in mouse models of thrombus [16]. In addition, it protects human umbilical vein endothelial cells (ECV-304) from the damage caused by nitrite and hydrogen peroxide [17]. These excellent properties indicate that subtilisin QK is a promising agent for the prevention and treatment of CVDs. However, toxicological data on subtilisin QK remain limited.

The aim of this study was to systematically evaluate the safety of oral subtilisin QK in Sprague–Dawley (SD) rats under good laboratory practice conditions, including acute and subchronic toxicity, and safety pharmacological studies. The results provide reliable toxicological data showing that subtilisin QK is a functional food or agent that may be used to prevent and treat CVDs.

## Methods

### Materials

Subtilisin QK was provided by Wuhan Zhenfu Pharmaceutical Co., Ltd. (Wuhan, China). Subtilisin QK was derived from *B. subtilis QK02* liquid fermentation. The fermentation broth was made into a powder by centrifugation, microfiltration, ultrafiltration concentration, and spray drying. Subtilisin QK activity was measured using a fibrin degradation assay developed by Japan Bio Science Laboratory Co., Ltd. (Walnut Creek, CA, USA). A spray-dried powder was tested for microbial limit, pH, biological activity, moisture, MW, protein content, and percentage. Analytical data from three batches of subtilisin QK are presented in Supplementary Table 1.

### Animals

Healthy female and male SD rats, aged 6–8 weeks, were purchased from Shanghai Jihui Experimental Animal Breeding Co., Ltd. (Shanghai, China), and all animals received 7 days of adaptive feeding. All SD rats were housed in a specific pathogen-free room (License No. SYXK (Su) 2016–0043). The room was maintained at 20.0–23.7 °C and a relative humidity of 49.4–55.1%, with a 12 h light/12 h dark cycle and minimum air change ≥15 times/h.

### Dose selection

In a previous study of nattokinase, the acute toxicity results showed that nattokinase did not cause apparent toxicity at a dose of 2000 mg/kg (49,400 FU/kg) [18]. Subtilisin QK and nattokinase are highly homologous, and belong to the subtilisin family. Therefore, we considered that subtilisin QK has low toxicity, and conducted a limit test. For some low-toxic subjects, the maximum dose method can be used (a single dose < 5000 mg/kg; National Food and Drug Administration, China, May 13, 2014). Since the maximum solubility of subtilisin QK is about 2500 FU/mL, and the maximum feeding volume is 40 mL/kg in a single day. The acute toxicity of subtilisin QK was determined using the maximum dose method in SD rats at a concentration of 10,0000 FU/kg (~ 4000 mg/kg). The results of our study on mouse tail vein thrombolysis showed that the lowest effective dose was 1250 FU/kg (the equivalent dose in rats is ~ 500 FU/kg). Therefore, in a subchronic toxicity test, the low, medium, and high doses were 2500, 7500, and 25,000 FU/kg, respectively, which were approximately 5, 15, and 50 times the equivalent dose in rats. In the pharmacology safety test, the low, medium, and high doses were 500, 1500, and 5000 FU/kg, respectively, which were approximately 1, 3, and 10 times the equivalent dose in rats.

### Acute toxicity study

Forty SD rats were randomly allocated to two groups (10 per sex). The study protocol was conducted in compliance with *Chinese technical guidelines for single-drug toxicity studies* and *Chinese general principles for technical evaluation of non-clinical safety of therapeutic biologic products* (National Food and Drug Administration, China, May 13, 2014). The treatment group was administered a single dose of 100,000 FU/kg subtilisin QK dissolved in saline, in a volume of 40 mL/kg. The control group was administered an equal volume of saline. Clinical signs of toxicity were closely monitored for 1, 3, and 6 h after administration, and all rats were monitored twice daily for 14 days. Body weights and food consumption of all animals were recorded before administration and on days 2, 7, and 14. On day 15, all animals were euthanized by carbon dioxide inhalation and were subjected to macroscopic examination. According to AVMA guidelines, carbon dioxide exposure is less likely to cause pain by a gradual-fill method. Therefore, euthanasia of rodents by inhaling carbon dioxide in their cages can help reduce stress and pain.

### Subchronic toxicity study

#### Experimental design

In total, 120 SD rats were randomly allocated to four groups (15 per sex). According to *OECD 407-Repeated Dose 28-day Oral Toxicity Study in Rodents* regulations, at least 10 animals (5 females and 5 males) should be used at each dose level. For the additional satellite group, 10 animals (5 per sex) were observed for reversibility, persistence, or delayed occurrence of toxic effects for at least 14 days post-treatment. To obtain sufficient oral toxicity and delayed toxicity data on rodents for 28 days prior to human use, we used 30 animals in each group (15 per sex). The study protocol was conducted in compliance with the *Chinese Technical Guidelines for Repeated-Dose Toxicity Studies* (National Food and Drug Administration, China, May 13, 2014). The dosing groups were administered single doses of 2500, 7500, and 25,000 FU/kg subtilisin QK dissolved in saline for 28 days, in a volume of 10 mL/kg. On day 29, the remaining animals (5 rats of each sex in each group) as the satellite group, were continuously monitored for a 28-day recovery period. Clinical signs of toxicity were monitored twice daily during the dosing period and during the recovery period. Food consumption of each animal was measured on days 7, 14, 21, 28, 35, 42, 49, and 56. Body weights of each animal were recorded on days 0, 7, 14, 21, 28, 35, 42, 49, and 56. Urinalysis, biochemical, hematological and coagulation parameters were conducted on days 28 and 56. Ophthalmological examination was conducted on days 0, 28 and 56, and pathological examination on days 28 and 56. Note: Day 0 represents before the dosing day, and day 1 represents the first day of dosing.

#### Ophthalmoscopy and urinalysis

Each animal underwent an eye examination with a slit lamp microscope and binocular indirect ophthalmoscope including eyelid, conjunctiva, cornea, sclera, iris, pupil, lens, vitreous, and fundus. Animals were placed in metabolic cages to collect fresh urine on days 28 and 56. After collection, urine samples were placed in a sample transport box and detected within 2 h. Urine samples of all animals were used for urinalysis with a urine chemistry analyzer (AUTION MAX AX-4280; ARKRAY Inc., Kyoto, Japan) including pH, color, turbidity, nitrite, glucose, specific gravity (SG), occult blood, protein, bilirubin (BIL), urobilinogen, and ketone.

#### Macroscopic findings and organ weights

All animals were anesthetized by intramuscular injection of Zoletil (75 mg/kg) at the end of the experiment. Blood was collected from the abdominal aorta after anesthesia, and then the animals were killed. During dissection, the general condition, body surface, thoracic cavity, abdominal cavity, pelvic cavity, and intracranial tissues/organs of the animal were examined, and all abnormal changes were recorded.

Organ weights were recorded (including brain, thymus, kidney, adrenals, thyroid, liver, spleen, testis, epididymis, uterus, and ovaries), and the relative organ weights were calculated by the following formula: relative organ weight = absolute organ weight (g)/body weight (g) × 100%.

#### Hematological, biochemical, and coagulation parameters

The blood samples collected in EDTA-K2-coated tubes were analyzed for hematology using an auto-hematology analyzer (XT-2000iV; Sysmex Co., Kobe, Japan) including white blood cell (WBC), WBC classification count (N, L, M, E, B) and classification percentage (N%, L%, M%, E%, B%), red blood cell, hemoglobin, hematocrit, mean corpuscular volume, mean corpuscular hemoglobin, mean corpuscular hemoglobin concentration, platelet, and reticulocyte count. The blood samples collected by inert separating gel coagulant tube were analyzed for blood biochemistry parameters including K^+^, Na^+^, Cl^−^, total BIL (TBIL), total protein, albumin, alanine aminotransferase, aspartate aminotransferase, alkaline phosphatase, gamma glutamyl transferase, urea, creatinine, glucose, triglyceride, and total cholesterol. Blood biochemistry was determined using an automatic biochemistry analyzer (c8000; Abbott Laboratories, Chicago, IL, USA). Na^+^, K^+^, and Cl^−^ were determined using an electrolyte analysis instrument (XI-931 T; Shenzhen City Kate Bio-Medical Electronics Co., Ltd., Shenzhen, China). The blood samples were collected in sodium citrate-coated tubes. Plasma was separated for coagulation detection using an automated coagulation analyzer (STAGO STA-R Evolution; Diagnostica Stago, Asnières-sur-Seine, France) including thrombin time (TT), prothrombin time, (PT) fibrinogen, and activated partial thromboplastin time (APTT).

#### Histopathology

Routine histopathological examinations were performed on related organs in the high-dose group (25,000 FU/kg) and control group (0 FU/kg). The brain, spinal cord, pituitary, thyroid, parathyroid glands, heart, aorta, trachea, lungs, main bronchi, salivary glands, esophagus, pancreas, stomach, small/large intestines, liver, gallbladder, kidneys, urinary bladder, testes, epididymis, uterus, oviduct, ovaries, cervix prostate, vaginal, sciatic nerve, skeletal muscle, adrenals, spleen, thymus, mesenteric lymph node, submandibular lymph node, bone marrow, skin, mammary gland, eye, and optic nerve were removed from each animal. The testis, epididymis, eyeball, and optic nerve were fixed in modified Davidson fixation solution, and the remaining tissues/organs were fixed in 10% neutral buffer formalin fixation solution. Then the sections were routinely processed, embedded in paraffin, sectioned, stained with hematoxylin and eosin, and examined under a microscope.

#### Safety pharmacology study

Forty SD rats were randomly allocated to four groups (5 per sex). The dosing groups were administered single doses of 500, 1500, and 5000 FU/kg subtilisin QK dissolved in saline, in a volume of 10 mL/kg. The control group was administered an equal volume of saline.

Functional observation battery (FOB) was recorded at 1 h prior to dosing and at 0.5, 1, 2, 4, 6, 8, and 24 h after dosing including in-cage observation (animal sleep, exercise, vertical hair, attacking caged animals, abnormal vocalization and grooming behavior), open field observation (position, autonomic activity, ataxia, hypotonia, convulsions, convulsions, stereotypes, vertical tail, vertical hair, erect, urination, respiration and panic reaction), and hand-held observation (location passive response, catalepsy, visual location, corneal response, auricular reflex, skin color, cyanosis, blinking, eyeball protrusion, pupillary light reflection, tear secretion, saliva secretion, tail-tail reaction, righting reflex, positive reflex, aggression/stress, abnormal vocalization, viscosity of bowel movements, and death and body temperature). Functional tests were recorded at 1 h prior to dosing and at 0.5, 1, 2, 4, 6, 8, and 24 h after dosing including spontaneous activity total distance, number of activities, and holding power. In the functional test, the universal spontaneous activity video analysis system (JLBehv-LAG-4; Shanghai Jinliang Software Technology Co. Ltd., Shanghai, China) was used to detect the spontaneous activity of the rats, and the number of spontaneous activities of the animals within 3 min was recorded at each time point. Rats were examined for limb grip using a grip tester (YLS-13A; Jinan Yiyan Technology Development Co. Ltd., Jinan, China). At least one adaptation training was performed on the autonomous animals before the test.

### Statistical analyses

Levene’s test was used to test the homogeneity of variance. If the variance was uniform (*p* > 0.01), the analysis of variance results were directly used to determine the statistical significance of the overall difference. If the variance was not uniform (*p* ≤ 0.01), the statistical significance of the overall difference was judged using results from Welch’s *t*-test. If the analysis of variance or Welch’s *t*-test results were statistically significant, multiple comparisons of differences between groups were performed using the Bonferroni test to further determine which differences were statistically significant. *P* < 0.05 was considered statistically significant.

## Results

### Acute toxicity study

In the acute toxicity study, no animals died in the control or 100,000 FU/kg dosing group. During the 14-day observation period, no adverse clinical signs were observed in the animals. Compared with the control group, there were no statistically significant differences in mean body weight and mean food consumption in the 100,000 FU/kg dosing group (Supplementary Table 2). Similarly, no significant pathology was observed upon macroscopic examination in all animals.

### Subchronic toxicity study

#### Clinical observations, body weight, and mean food consumption

**Fig. 1:**
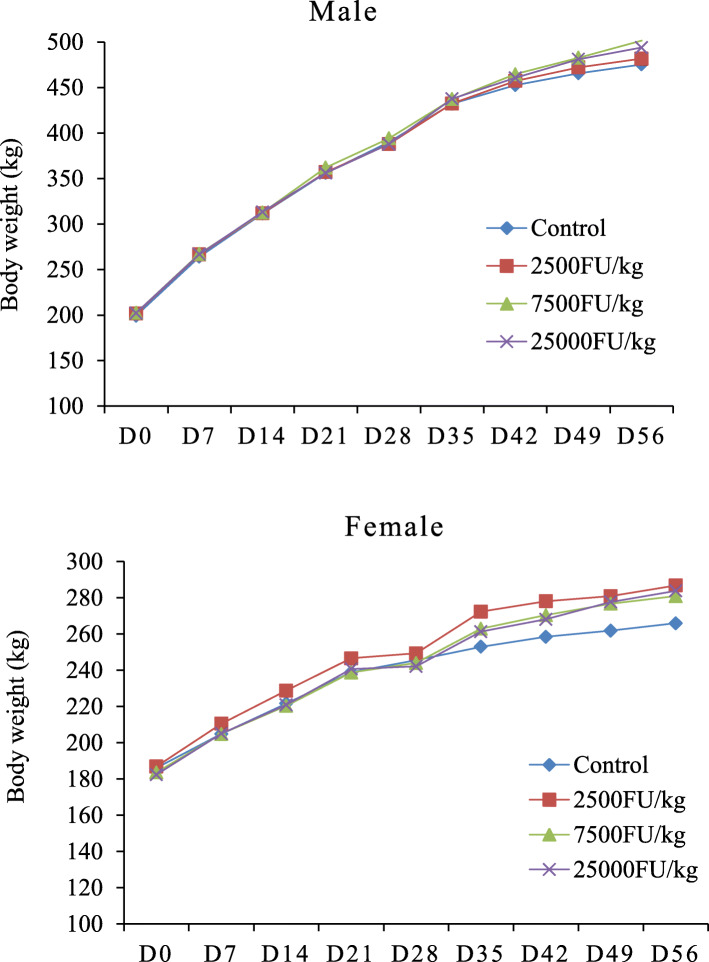
Mean body weight of Sprague Dawley rats administration with subtilisin QK for 28-day and 28-day recovery

**Fig. 2:**
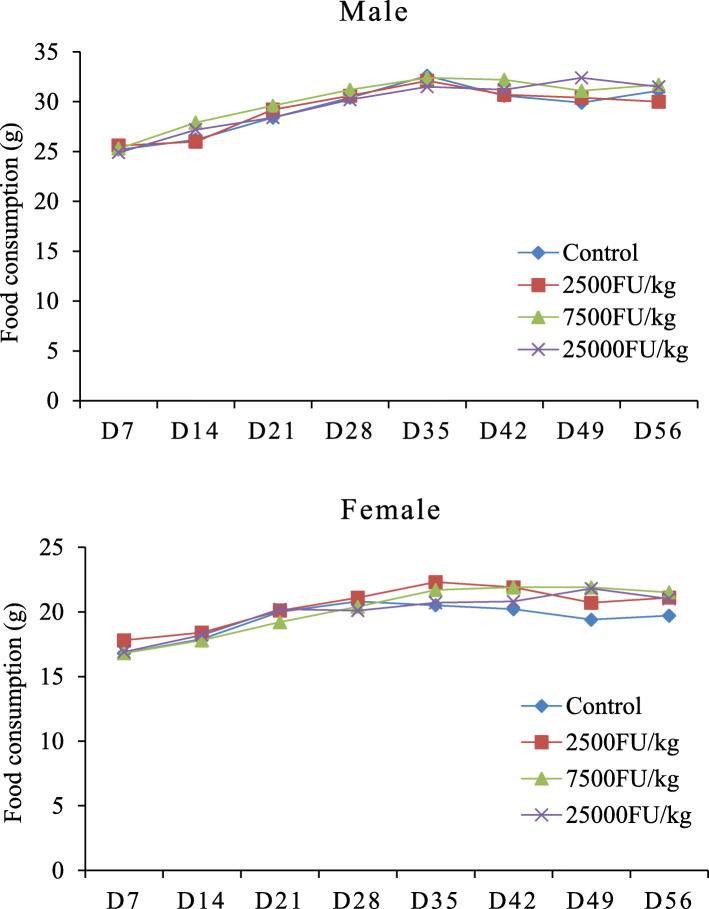
Mean food consumption of Sprague Dawley rats administration with subtilisin QK for 28-day and 28-day recovery

During the dosing period of 28 days and recovery period of 28 days, no animal died and no abnormal behavior or overt signs of toxicity were observed in the treatment groups. Compared with the control group, there were no statistically significant differences in mean body weight and mean food consumption in the treatment groups (Figs. 1 and 2).

#### Ophthalmoscopy and urinalysis

There were no obvious ophthalmoscopic abnormalities in the animals (data not shown). With the exception of SG in mid-dose females (7500 FU/kg-day) being higher than that the controls (*p* < 0.05), there were no significant differences in the urinalysis results in the dosing and control groups. Due to the lack of a dose–response relationship, this finding was not considered treatment-related. (Table 1).

**Table 1:** Urinalysis results of Sprague Dawley rats administration with subtilisin QK for 28 days and 28 days recovery

		Groups	PH	SG	TURB	NIT	GLU	BLD	PRO	BIL	URO	KET	LEU
D_28_	male	control	8.6 ± 0.2	1.030 ± 0.010	2(−); 8(+ 1)	8(−);1(+ 2)	10(−)	10(−)	7(+ 1); 2(+ 2); 1(+ 4)	10(−)	7(−); 3(+ 1)	5(−); 4(+−); 1(+ 1)	8(−); 1(+ 1); 1(+ 2)
*n* = 15		2500 FU/kg	8.5 ± 0.0	1.031 ± 0.014	1(−); 8(+ 1); 1(+ 2)	8(−); 1(+ 1); 1(+ 2)	10(−)	10(−)	6(+ 1); 3(+ 2); 1(+ 4)	10(−)	7(−); 3(+ 1)	5(−); 5(+−)	7(−); 2(+ 1); 1(+ 2)
		7500 FU/kg	8.4 ± 0.2	1.021 ± 0.013	3(−); 5(+ 1); 2(+ 2)	4(−); 6(+ 1)	10(−)	10(−)	9(+ 1); 1(+ 2)	10(−)	9(−); 1(+ 1)	9(−); 1(+ 1)	8(−); 2(+ 1)
		25,000 FU/kg	8.4 ± 0.2	1.030 ± 0.006	6(+ 1); 4(+ 2)	1(−); 7(+ 1); 2(+ 2)	10(−)	10(−)	7(+ 1); 3(+ 2)	10(−)	9(−); 1(+ 1)	7(−); 3(+−)	8(−); 2(+ 1)
	female	control	8.5 ± 0.0	1.012 ± 0.003	1(−); 9(+ 1)	1(−); 4(+ 1); 5(+ 2)	10(−)	10(−)	7(+ 1); 3(+ 2)	10(−)	10(−)	10(−)	9(−); 1(+ 1)
		2500 FU/kg	8.5 ± 0.2	1.012 ± 0.003	2(−); 8(+ 1)	4(−); 3(+ 1); 3(+ 2)	10(−)	10(−)	10(−)	10(−)	10(−)	10(−)	9(−); 1(+ 1)
		7500 FU/kg	8.4 ± 0.2	1.015 ± 0.005	2(−); 8(+ 1)	1(−); 5(+ 1); 4(+ 2)	10(−)	9(−); 1(+ 2)	7(+ 1); 3(+ 2)	10(−)	10(−)	10(−)	10(−)
		25,000 FU/kg	8.4 ± 0.2	1.019 ± 0.008	2(−); 6(+ 1); 2(+ 2)	2(−); 5(+ 1); 3(+ 2)	10(−)	10(−)	4(+ 1); 6(+ 2)	10(−)	9(−); 1(+ 1)	10(−)	8(−); 2(+ 1)
D_56_	male	control	8.5 ± 0.0	1.018 ± 0.006	5(+ 1)	1(−); 1(+ 1); 3(+ 2)	5(−)	4(−); 1(+ 1)	5(+ 1)	5(−)	5(−)	3(−); 2(+−)	4(−); 1(+ 1)
*n* = 5		2500 FU/kg	8.5 ± 0.0	1.020 ± 0.006	2(−); 3(+ 1)	1(−); 4(+ 1)	5(−)	3(−); 2(+ 1)	4(+−); 1(+ 1)	5(−)	5(−)	3(−); 1(+−); 1(+ 1)	5(−)
		7500 FU/kg	8.5 ± 0.0	1.028 ± 0.002*	5(+ 1)	2(+ 1); 3(+ 2)	5(−)	5(−)	2(+ 1); 3(+ 2)	5(−)	5(−)	1(−); 4(+−)	2(−); 3(+ 1)
		25,000 FU/kg	8.5 ± 0.0	1.017 ± 0.004	1(−); 2(+ 1); 2(+ 2)	1(+ 1); 4(+ 2)	5(−)	4(−); 1(+ 1)	1(+−); 2(+ 1); 3(+ 2)	5(−)	5(−)	5(−)	5(−)
	female	control	8.0 ± 0.9	1.014 ± 0.006	2(−); 3(+ 1)	2(−); 3(+ 1)	5(−)	5(−)	4(−); 1(+−);	5(−)	5(−)	5(−)	5(−)
		2500 FU/kg	8.2 ± 0.3	1.014 ± 0.003	4(−); 1(+ 1)	3(−); 2(+ 1)	5(−)	5(−)	5(−)	5(−)	5(−)	5(−)	5(−)
		7500 FU/kg	7.9 ± 0.2	1.011 ± 0.004	1(−); 4(+ 1)	5(+ 1)	5(−)	4(−); 1(+−);	2(−); 3(+−);	5(−)	5(−)	5(−)	5(−)
		25,000 FU/kg	7.6 ± 0.8	1.011 ± 0.004	4(−); 1(+ 1)	4(−); 1(+ 1)	5(−)	5(−)	4(−); 1(+−);	5(−)	5(−)	5(−)	5(−)

#### Hematological, blood biochemical, and coagulation parameters

Results of the hematology analyses are shown in Supplementary Table 3. The mean corpuscular hemoglobin (MCH) was higher than the corresponding controls in mid-dose males (7500 FU/kg-day; *p* < 0.05) on day 28; the mean corpuscular hemoglobin concentration (MCHC) was lower than the corresponding controls in high-dose males in (25,000 FU/kg-day; *p* < 0.05). However, these changes lacked a dose–response relationship and thus were considered to be unrelated to treatment.

Results of the clinical chemistry analyses are shown in Table 2. The mean concentration of K^+^ was higher than the corresponding controls in mid-dose females (7500 FU/kg-day; *p* < 0.05) on day 28 and mid-dose males (7500 FU/kg-day; *p* < 0.05) on day 56. The mean concentration of TBIL was higher than the control in mid-dose males (7500 FU/kg-day; *p* < 0.05) on day 28. There were also statistically significant differences in the mean concentration of urea in low-dose males (2500 FU/kg-day; *p* < 0.05), mid-dose males (7500 FU/kg-day; *p* < 0.05), and high-dose males (25,000 FU/kg-day; *p* < 0.05) on day 56. However, these differences were considered unrelated to treatment due to being within their corresponding normal ranges (Table 2).

**Table 2:** Biochemical parameters of Sprague Dawley rats administration with subtilisin QK for 28 days and 28 days recovery

Day		Normal range	Male				Female			
			Control	2500 FU/kg	7500 FU/kg	25,000 FU/kg	Control	2500 FU/kg	7500 FU/kg	25,000 FU/kg
D_28_	TBIL (umol/L)	0.3–1.2	1.04 ± 0.14	1.04 ± 0.23	1.25 ± 0.14*	1.22 ± 0.13	1.90 ± 0.27	1.94 ± 0.42	2.13 ± 0.35	2.19 ± 0.26
*n* = 15	TP (g/L)	65–82	58.38 ± 2.80	56.00 ± 2.06	57.19 ± 2.00	58.07 ± 2.63	62.59 ± 2.09	60.56 ± 3.18	61.45 ± 3.78	61.07 ± 2.18
	ALB (g/L)	27–55	33.76 ± 1.55	32.98 ± 1.29	33.26 ± 0.69	33.51 ± 1.10	36.74 ± 1.34	36.10 ± 2.14	36.16 ± 2.12	36.37 ± 1.72
	GLOB (g/L)	18–30	24.62 ± 1.47	23.02 ± 0.92	23.93 ± 1.76	24.56 ± 1.62	25.85 ± 1.36	24.46 ± 1.46	25.29 ± 1.85	24.70 ± 0.66
	ALT (U/L)	5–40	34.90 ± 3.51	33.60 ± 3.78	33.40 ± 4.12	32.70 ± 3.13	26.20 ± 4.02	35.33 ± 24.88	25.90 ± 4.72	24.00 ± 2.87
	AST (U/L)	100–140	124.30 ± 20.66	136.90 ± 29.06	133.70 ± 17.61	154.70 ± 38.69	124.50 ± 28.38	138.33 ± 77.26	117.20 ± 28.18	126.00 ± 18.48
	ALP (U/L)	104–338	181.60 ± 39.16	172.50 ± 25.31	156.00 ± 33.54	171.50 ± 29.44	84.60 ± 16.00	83.00 ± 17.90	86.80 ± 31.85	83.80 ± 26.81
	GGT (U/L)	< 0.79	0.30 ± 0.48	0.20 ± 0.42	0.10 ± 0.32	0.40 ± 0.52	0.40 ± 0.52	0.56 ± 0.53	0.10 ± 0.32	0.50 ± 0.53
	UREA (mmol/L)	2.7–7.0	5.28 ± 0.62	5.18 ± 0.47	5.12 ± 0.55	4.92 ± 0.60	5.98 ± 1.12	6.03 ± 0.94	6.02 ± 1.03	5.75 ± 0.96
	CREA (umol/L)	20–80	19.47 ± 2.19	20.87 ± 1.90	19.34 ± 2.53	19.37 ± 1.43	24.53 ± 4.11	24.21 ± 3.90	24.16 ± 3.18	25.14 ± 2.47
	GLU (mmol/L)	7–10.9	7.14 ± 0.78	7.09 ± 0.99	6.14 ± 0.71	7.28 ± 0.98	5.81 ± 0.77	6.19 ± 0.97	6.29 ± 0.71	5.80 ± 0.52
	TG (mmol/L)	0.2–1.4	0.31 ± 0.10	0.24 ± 0.08	0.27 ± 0.10	0.29 ± 0.14	0.21 ± 0.06	0.19 ± 0.05	0.20 ± 0.07	0.18 ± 0.08
	CHOL (mmol/L)	0.4–1.3	1.43 ± 0.20	1.26 ± 0.22	1.26 ± 0.23	1.36 ± 0.29	1.66 ± 0.31	1.67 ± 0.55	1.60 ± 0.42	1.27 ± 0.34
	K^+^ (mmol/L)	3.5–5.0	4.93 ± 0.42	4.89 ± 0.40	4.88 ± 0.41	5.00 ± 0.37	4.34 ± 0.35	4.50 ± 0.28	4.87 ± 0.46*	4.43 ± 0.30
	Na^+^ (mmol/L)	135–145	141.30 ± 1.95	141.80 ± 1.32	141.50 ± 1.58	141.60 ± 1.51	140.30 ± 0.67	140.56 ± 1.01	140.80 ± 1.40	140.50 ± 1.18
	Cl^−^ (mmol/L)	98–108	103.90 ± 1.66	105.10 ± 0.88	104.60 ± 1.26	104.20 ± 2.66	105.90 ± 0.74	104.78 ± 1.39	105.20 ± 2.35	105.30 ± 1.64
D_56_	TBIL (umol/L)	0.3–1.2	1.74 ± 0.30	1.64 ± 0.29	1.56 ± 0.30	1.64 ± 0.34	1.74 ± 0.35	1.62 ± 0.28	1.96 ± 0.35	2.34 ± 0.70
*n* = 5	TP (g/L)	65–82	59.30 ± 2.87	59.64 ± 3.11	60.54 ± 3.26	59.74 ± 1.92	65.60 ± 3.50	63.90 ± 2.03	65.96 ± 2.54	68.84 ± 1.36
	ALB (g/L)	27–55	33.30 ± 1.14	33.54 ± 1.43	34.30 ± 1.48	33.74 ± 0.34	38.46 ± 2.82	37.64 ± 0.96	38.56 ± 1.45	39.64 ± 0.59
	GLOB (g/L)	18–30	26.00 ± 1.98	26.10 ± 1.75	26.24 ± 1.92	26.00 ± 1.69	27.14 ± 1.51	26.26 ± 1.11	27.40 ± 1.56	29.20 ± 1.37
	ALT (U/L)	5–40	46.20 ± 7.33	46.40 ± 7.30	39.00 ± 7.38	38.60 ± 1.14	43.60 ± 11.04	35.20 ± 6.98	32.60 ± 5.46	39.00 ± 4.06
	AST (U/L)	100–140	160.80 ± 17.91	159.60 ± 28.92	143.20 ± 27.74	159.40 ± 21.04	136.00 ± 40.63	123.00 ± 15.38	121.20 ± 24.16	144.00 ± 55.19
	ALP (U/L)	104–338	114.80 ± 25.01	118.80 ± 22.20	115.00 ± 23.29	99.00 ± 16.84	61.60 ± 13.61	61.40 ± 12.93	61.80 ± 10.03	51.80 ± 12.81
	GGT (U/L)	< 0.79	0.20 ± 0.45	0.80 ± 0.45	0.40 ± 0.55	0.60 ± 0.55	0.40 ± 0.55	1.20 ± 0.45	0.80 ± 0.45	0.60 ± 0.55
	UREA (mmol/L)	2.7–7.0	7.14 ± 0.67	5.91 ± 0.64*	5.91 ± 0.65*	5.92 ± 0.52*	6.72 ± 0.67	7.38 ± 0.99	6.45 ± 1.31	6.93 ± 1.53
	CREA (umol/L)	20–80	23.74 ± 2.59	23.62 ± 2.67	24.60 ± 3.17	25.94 ± 1.23	29.58 ± 4.52	29.82 ± 4.03	28.48 ± 5.00	28.30 ± 5.05
	GLU (mmol/L)	7–10.9	8.47 ± 0.91	8.24 ± 2.11	7.67 ± 0.93	7.43 ± 0.50	8.48 ± 1.41	9.14 ± 1.46	8.25 ± 0.61	7.30 ± 0.21
	TG (mmol/L)	0.2–1.4	0.35 ± 0.28	0.32 ± 0.11	0.29 ± 0.13	0.24 ± 0.14	0.21 ± 0.06	0.25 ± 0.09	0.27 ± 0.11	0.21 ± 0.04
	TCHO (mmol/L)	0.4–1.3	1.34 ± 0.29	1.22 ± 0.40	1.35 ± 0.34	1.34 ± 0.19	1.52 ± 0.20	1.46 ± 0.45	1.49 ± 0.39	1.87 ± 0.45
	K^+^ (mmol/L)	3.5–5.0	5.04 ± 0.27	4.88 ± 0.18	4.50 ± 0.10*	4.82 ± 0.27	4.16 ± 0.27	4.10 ± 0.24	4.54 ± 0.21	4.58 ± 0.16
	Na^+^ (mmol/L)	135–145	140.80 ± 0.84	141.20 ± 1.30	141.80 ± 1.64	141.60 ± 0.55	140.00 ± 1.00	140.20 ± 0.84	139.80 ± 0.84	140.20 ± 0.84
	Cl^−^ (mmol/L)	98–108	103.00 ± 1.22	104.20 ± 1.30	105.20 ± 1.30	104.20 ± 1.10	105.80 ± 0.45	106.20 ± 0.84	106.40 ± 0.89	106.40 ± 1.67

*TBIL* Total bilirubin, *TP* Total protein, *ALB* Albumin, *ALT* Alanine aminotransferase, *AST* Aspartate aminotransferase, *ALP* Alkaline phosphatase, *GGT* Gamma-glutamyl transferase, *UREA* Urea, *CREA* Creatinine, *GLU* Glucose, *TG* Triglyceride, *TCHO* Total cholesterol, *K* Potassium, *Cl* Chloride, *Na* Sodium

*p < 0.05

Results of the coagulation function analyses are shown in Table 3. On day 28, the APTT was lower than the corresponding controls in mid-dose males (7500 FU/kg-day; *p* < 0.05) and high-dose males (25,000 FU/kg-day; *p* < 0.05). The PT was higher than the control in high-dose females (25,000 FU/kg-day; *p* < 0.05) on day 28, and lower than the controls in mid-dose females (7500 FU/kg-day; *p* < 0.05) and high-dose females (25,000 FU/kg-day; *p* < 0.05) on day 56. Due to being within their corresponding normal ranges, these findings were not considered treatment-related.

**Table 3:** Coagulation parameters of Sprague Dawley rats administration with subtilisin QK for 28 days and 28 days recovery

		Male				Female			
		Control	2500 FU/kg	7500 FU/kg	25,000 FU/kg	Control	2500 FU/kg	7500 FU/kg	25,000 FU/kg
D_28_	PT	16.40 ± 0.63	16.42 ± 0.46	16.72 ± 0.56	16.81 ± 0.47	15.36 ± 0.40	16.25 ± 0.92*	15.85 ± 0.71	15.98 ± 0.50
	APTT	23.74 ± 2.82	21.97 ± 2.67	19.81 ± 1.65*	19.96 ± 2.22*	20.12 ± 2.31	19.08 ± 1.98	20.17 ± 1.87	17.79 ± 2.18
	TT	32.47 ± 1.48	32.39 ± 2.18	32.44 ± 1.83	32.38 ± 1.01	32.46 ± 2.29	33.13 ± 1.82	34.03 ± 1.05	31.74 ± 1.27
	FIB	3.01 ± 0.21	2.93 ± 0.22	3.10 ± 0.49	3.18 ± 0.32	2.50 ± 0.24	2.64 ± 0.33	2.61 ± 0.18	2.48 ± 0.26
D_56_	PT	16.64 ± 0.50	17.14 ± 1.02	17.52 ± 1.13	16.88 ± 0.91	17.10 ± 0.66	16.62 ± 0.41	16.10 ± 0.39*	16.02 ± 0.54*
	APTT	23.72 ± 2.30	23.26 ± 0.57	25.78 ± 4.29	23.26 ± 1.79	19.78 ± 1.08	18.98 ± 1.14	20.36 ± 2.22	20.86 ± 1.80
	TT	36.68 ± 0.96	35.28 ± 1.84	35.78 ± 2.02	35.66 ± 1.14	36.94 ± 1.27	35.38 ± 1.03	34.98 ± 1.58	36.68 ± 1.53
	FIB	2.69 ± 0.15	2.87 ± 0.24	2.95 ± 0.19	2.91 ± 0.26	2.23 ± 0.32	2.28 ± 0.18	2.43 ± 0.09	2.21 ± 0.06

*p < 0.05. *TT* Thrombin time, *PT* Prothrombin time, *FIB* Fibrinogen, *APTT* Activated partial thromboplastin time

#### Organ weights and macroscopic findings

There was a statistically significant difference in mean absolute and relative epididymis weight (*p* < 0.05) in low-dose males (2500 FU/kg-day) relative to the controls in the satellite group; however, these changes were only seen in the low-dose males. Therefore, these findings were not considered related to subtilisin QK treatment (Tables 4 and 5). In the macroscopic examination, there were no macroscopic signs of pathology in all groups.

**Table 4:** Absolute organ weights of Sprague Dawley rats administration with subtilisin QK for 28 days and 28 days recovery

		Male				Female			
		Control	2500 FU/kg	7500 FU/kg	25,000 FU/kg	Control	2500 FU/kg	7500 FU/kg	25,000 FU/kg
D_28_	heart	1.361 ± 0.157	1.284 ± 0.100	1.353 ± 0.161	1.290 ± 0.103	0.899 ± 0.109	0.877 ± 0.129	0.979 ± 0.264	0.875 ± 0.076
*n* = 10	liver	10.591 ± 1.201	10.509 ± 1.030	10.769 ± 1.015	10.920 ± 1.114	6.804 ± 0.701	6.636 ± 0.708	6.520 ± 0.987	6.510 ± 0.541
	spleen	0.712 ± 0.160	0.725 ± 0.134	0.714 ± 0.085	0.732 ± 0.128	0.500 ± 0.069	0.483 ± 0.044	0.514 ± 0.051	0.473 ± 0.038
	kidenys	2.789 ± 0.198	2.600 ± 0.237	2.768 ± 0.163	2.695 ± 0.208	1.747 ± 0.204	1.743 ± 0.154	1.692 ± 0.221	1.709 ± 0.170
	thymus	0.486 ± 0.113	0.469 ± 0.073	0.497 ± 0.123	0.428 ± 0.077	0.385 ± 0.108	0.353 ± 0.070	0.337 ± 0.091	0.375 ± 0.073
	adrenals	0.072 ± 0.008	0.072 ± 0.013	0.073 ± 0.007	0.075 ± 0.013	0.086 ± 0.010	0.083 ± 0.015	0.077 ± 0.016	0.080 ± 0.016
	testis	3.041 ± 0.368	2.903 ± 0.242	2.986 ± 0.108	3.003 ± 0.261	–	–	–	–
	epididymis	0.902 ± 0.097	0.887 ± 0.078	0.920 ± 0.074	0.894 ± 0.074	–	–	–	–
	uterus	–	–	–	–	0.518 ± 0.074	0.527 ± 0.169	0.491 ± 0.061	0.621 ± 0.184
	ovaries	–	–	–	–	0.090 ± 0.014	0.094 ± 0.032	0.092 ± 0.011	0.096 ± 0.021
	brain	1.967 ± 0.097	1.986 ± 0.062	1.958 ± 0.077	1.984 ± 0.081	1.909 ± 0.080	1.889 ± 0.072	1.905 ± 0.098	1.863 ± 0.081
D_56_	heart	1.605 ± 0.203	1.535 ± 0.074	1.661 ± 0.244	1.486 ± 0.016	0.915 ± 0.059	0.984 ± 0.102	1.065 ± 0.134	0.959 ± 0.058
*n* = 5	liver	13.193 ± 1.495	12.676 ± 0.890	13.032 ± 1.653	12.452 ± 1.449	7.476 ± 1.002	7.664 ± 0.660	7.630 ± 1.037	7.374 ± 0.486
	spleen	0.793 ± 0.087	0.732 ± 0.091	0.814 ± 0.059	0.798 ± 0.104	0.535 ± 0.047	0.558 ± 0.048	0.518 ± 0.074	0.557 ± 0.112
	kidenys	3.447 ± 0.331	3.327 ± 0.208	3.207 ± 0.466	3.409 ± 0.444	1.754 ± 0.079	1.925 ± 0.202	1.931 ± 0.240	1.883 ± 0.164
	thymus	0.326 ± 0.071	0.341 ± 0.114	0.402 ± 0.067	0.402 ± 0.026	0.259 ± 0.051	0.297 ± 0.075	0.318 ± 0.045	0.252 ± 0.070
	adrenals	0.074 ± 0.011	0.076 ± 0.012	0.067 ± 0.009	0.075 ± 0.018	0.081 ± 0.010	0.077 ± 0.008	0.084 ± 0.012	0.080 ± 0.009
	testis	3.329 ± 0.132	3.281 ± 0.158	3.553 ± 0.501	3.271 ± 0.152	–	–	–	–
	epididymis	1.426 ± 0.108	1.232 ± 0.041*	1.391 ± 0.142	1.403 ± 0.070	–	–	–	–
	uterus	–	–	–	–	0.590 ± 0.174	0.510 ± 0.047	0.631 ± 0.034	0.653 ± 0.187
	ovaries	–	–	–	–	0.091 ± 0.012	0.094 ± 0.015	0.086 ± 0.023	0.077 ± 0.012
	brain	2.139 ± 0.092	2.140 ± 0.076	2.153 ± 0.095	2.148 ± 0.045	1.948 ± 0.037	1.964 ± 0.052	1.936 ± 0.129	1.918 ± 0.072

*p < 0.05

**Table 5:** Relative mean organ weights of Sprague Dawley rats administration with subtilisin QK for 28 days and 28 days recovery

		Male				Female			
		Control	2500 FU/kg	7500 FU/kg	25,000 FU/kg	Control	2500 FU/kg	7500 FU/kg	25,000 FU/kg
D_28_	heart	0.354 ± 0.024	0.338 ± 0.019	0.351 ± 0.031	0.340 ± 0.021	0.368 ± 0.029	0.359 ± 0.032	0.406 ± 0.109	0.376 ± 0.036
*n* = 10	liver	2.753 ± 0.180	2.759 ± 0.167	2.798 ± 0.151	2.875 ± 0.199	2.787 ± 0.137	2.721 ± 0.185	2.691 ± 0.230	2.786 ± 0.157
	spleen	0.185 ± 0.037	0.190 ± 0.030	0.186 ± 0.021	0.192 ± 0.026	0.205 ± 0.020	0.198 ± 0.014	0.214 ± 0.024	0.203 ± 0.023
	kidenys	0.727 ± 0.033	0.683 ± 0.044	0.721 ± 0.046	0.710 ± 0.034	0.715 ± 0.042	0.716 ± 0.047	0.699 ± 0.048	0.731 ± 0.052
	thymus	0.127 ± 0.028	0.123 ± 0.018	0.129 ± 0.029	0.112 ± 0.018	0.156 ± 0.033	0.145 ± 0.028	0.139 ± 0.032	0.160 ± 0.029
	adrenals	0.019 ± 0.003	0.019 ± 0.004	0.019 ± 0.003	0.020 ± 0.003	0.035 ± 0.004	0.034 ± 0.005	0.032 ± 0.006	0.034 ± 0.005
	testis	0.792 ± 0.080	0.765 ± 0.070	0.778 ± 0.043	0.792 ± 0.059	–	–	–	–
	epididymis	0.235 ± 0.022	0.233 ± 0.020	0.240 ± 0.026	0.237 ± 0.025	–	–	–	–
	uterus	–	–	–	–	0.214 ± 0.036	0.215 ± 0.060	0.205 ± 0.029	0.268 ± 0.087
	ovaries	–	–	–	–	0.037 ± 0.006	0.039 ± 0.014	0.038 ± 0.006	0.041 ± 0.008
	brain	0.513 ± 0.018	0.524 ± 0.037	0.510 ± 0.031	0.524 ± 0.039	0.787 ± 0.060	0.777 ± 0.042	0.793 ± 0.063	0.800 ± 0.061
D_56_	heart	0.337 ± 0.020	0.319 ± 0.023	0.330 ± 0.021	0.302 ± 0.020	0.345 ± 0.027	0.344 ± 0.040	0.378 ± 0.027	0.338 ± 0.016
*n* = 5	liver	2.771 ± 0.151	2.633 ± 0.198	2.596 ± 0.202	2.515 ± 0.171	2.820 ± 0.450	2.675 ± 0.190	2.711 ± 0.226	2.600 ± 0.135
	spleen	0.168 ± 0.026	0.152 ± 0.018	0.163 ± 0.022	0.161 ± 0.019	0.202 ± 0.021	0.195 ± 0.012	0.184 ± 0.014	0.197 ± 0.039
	kidenys	0.725 ± 0.036	0.690 ± 0.036	0.637 ± 0.049	0.689 ± 0.068	0.661 ± 0.044	0.670 ± 0.041	0.687 ± 0.068	0.663 ± 0.033
	thymus	0.069 ± 0.013	0.071 ± 0.024	0.081 ± 0.017	0.082 ± 0.009	0.098 ± 0.020	0.103 ± 0.025	0.114 ± 0.015	0.088 ± 0.022
	adrenals	0.016 ± 0.003	0.016 ± 0.002	0.014 ± 0.002	0.015 ± 0.003	0.030 ± 0.003	0.027 ± 0.002	0.030 ± 0.007	0.028 ± 0.003
	testis	0.703 ± 0.057	0.682 ± 0.045	0.708 ± 0.066	0.664 ± 0.055	–	–	–	–
	epididymis	0.301 ± 0.034	0.256 ± 0.013*	0.277 ± 0.008	0.284 ± 0.017	–	–	–	–
	uterus	–	–	–	–	0.223 ± 0.068	0.178 ± 0.014	0.226 ± 0.019	0.230 ± 0.067
	ovaries	–	–	–	–	0.034 ± 0.005	0.033 ± 0.005	0.031 ± 0.007	0.027 ± 0.005
	brain	0.453 ± 0.047	0.444 ± 0.007	0.431 ± 0.026	0.436 ± 0.032	0.734 ± 0.036	0.687 ± 0.039	0.692 ± 0.059	0.678 ± 0.037

*p < 0.05

#### Histopathology

Representative photographs of organs from a female and a male for the control (0 FU/kg) and high-dose (25,000 FU/kg) groups are shown in Supplementary Fig. 1. The tissues were stained with hematoxylin and eosin. The histopathology results for the control (0 FU/kg) and high-dose (25,000 FU/kg) groups are shown in Table 6. The most common observations were pulmonary hemorrhage of the lung (3/15 control males and 1/15 high-dose males; 1/15 control females and 1/15 high-dose females) and renal tubular basophilia (5/15 control males and 3/15 high-dose males; 1/15 control females and 2/15 high-dose females). Since the pathological changes were spontaneous or sporadic background lesions of SD rats between the control and high-dose groups, there was no significant relationship with subtilisin QK.

**Table 6:** Main histopathology findings of Sprague Dawley rats administration with subtilisin QK for 28-day and 28-day recovery

	Male				Female			
	Control		25,000 FU/kg		Control		25,000 FU/kg	
	D_28_	D_56_	D_28_	D_56_	D_28_	D_56_	D_28_	D_56_
Lung, Pulmonary hemorrhage, limitations	3/10	0/5	1/10	0/5	1/10	0/5	1/10	0/5
Pancreas, Leaflet atrophy, limitations (slight)	0/10	0/5	1/10	0/5	0/10	0/5	0/10	0/5
Sialaden, Granular tube eosinophilic particle reduction (slight)	0/10	0/5	1/10	0/5	0/10	0/5	0/10	0/5
Ileum, Pei’s mineralization stove (slight)	0/10	0/5	2/10	0/5	0/10	0/5	0/10	0/5
Liver, Inflammatory cell infiltration, focal (slight)	1/10	0/5	1/10	1/5	0/10	1/5	0/10	0/5
Liver, Hepatic steatosis, lobular periphery	0/10	0/5	0/10	0/5	1/10	0/5	2/10	0/5
Kidney, Tubular basophilic lesion, focal (slight)	3/10	2/5	2/10	1/5	1/10	0/5	1/10	1/5
Bladder, mesenteric mineralization (slight)	0/10	0/5	1/10	0/5	0/10	0/5	0/10	0/5
Testicular, submucosal and interstitial hemorrhage, one side	0/10	0/5	1/10	0/5	–	–	–	–
Prostatic, interstitial lymphocytic infiltration	0/10	1/5	1/10	2/5	–	–	–	–
Eye, Retinal dysplasia (slight)	1/10	0/5	1/10	0/5	0/10	0/5	2/10	0/5
Harrington gland, Lymphocyte infiltration, one side (slight)	1/10	0/5	0/10	0/5	0/10	0/5	0/10	0/5

#### Safety pharmacology study

In the safety pharmacology test, there was no animal death. Compared with before the self-administration and the control group, there were no significant changes in FOB (cage observation, open field observation, and hand-held observation) in the dosing groups. Similarly, there were no statistically significant differences in spontaneous activity and total distance, number of activities, and holding power between the dosing and control groups. Those results showed that a single oral dose of 5000 FU/kg subtilisin QK had no effect on the rat central nervous system (Supplementary Table 4).

## Discussion

Heart disease and stroke account for about 85% of all deaths from CVDs (https://www.who.int/en/news-room/fact-sheets/detail/cardiovascular-diseases-(cvds)). However, safe and effective agents for CVD prevention and treatment are very limited. Oral anticoagulants such as aspirin can greatly reduce the formation of thrombus and decrease the risk of CVDs. However, they also markedly increase the risk of bleeding [19]. In addition, emergency treatment of CVDs with t-PA greatly increases the risk of bleeding [20]. Therefore, there is an urgent need for safer and more effective drugs to replace current treatments. Subtilisins including subtilisin QK, nattokinase, and subtilisin DEF are a class of serine proteases secreted by *B. subtilis* with excellent thrombolysis functions [4, 15, 21]. Nattokinase was shown to dissolve the thrombus in rat model of thrombosis [22]. In addition, nattokinase also possesses anticoagulant, antihypertensive and antiatherosclerotic properties in rats [23–25]. Clinical trials have shown that oral nattokinase can reduce the risk of CVD and Alzheimer’s disease [25, 26]. Subtilisin QK not only has strong thrombolytic capacity in mouse models of thrombus but also protects human umbilical vein endothelial cells (ECV-304) from the damage caused by nitrite and hydrogen peroxide [16, 17]. Therefore, subtilisins are promising agents for CVD prevention with significant development potential. However, toxicological data on subtilisins remain limited.

Here, subtilisin QK exhibited high safety in acute toxicity, 28-day subchronic toxicity, and safety pharmacological studies. First, an oral acute toxicity study of subtilisin QK in SD rats was conducted. Nattokinase has been reported to have no significant adverse effects on SD rats at a dose of 2000 mg/kg (49,400 FU/kg) [18]. The maximum daily tolerated dose of subtilisin QK in SD rats is 100,000 FU/kg (~ 4000 mg/kg), which is approximately two times more compared to the acute toxicity of nattokinase and 600 times more compared to the recommended daily dose for humans. Despite this, subtilisin QK did not cause death and obvious pathological changes. The results further indicated that subtilisins have a good safety profile.

Considering the long-term intake period in terms of preventing CVD, a comprehensive 28-day subchronic toxic evaluation of subtilisin QK was conducted. SD rats were administered daily oral doses of 2500, 7500, and 25,000 FU/kg subtilisin QK for 28 days to observe the toxicity and dose–response relationship of subtilisin QK. At the same time, a satellite group was established to observe the reversibility, persistence, or delayed toxic effects for 28 days post-treatment. The hematological results showed that there was a statistically significant increase in the MCH of 7500 FU/kg dose males on day 28 (*p* < 0.05), and a significant decrease in the MCHC of 25,000 FU/kg males on day 28 (*p* < 0.05) compared to the control group. Due to the lack of dose–response relationship and adverse effects in the satellite group, we concluded that the slight increase in MCH and decrease in MCHC were non-adverse (Supplementary Table 3). The blood biochemistry showed that K^+^ was increased in 7500 FU/kg females on day 28 (*p* < 0.05) and was slightly decreased in 7500 FU/kg males in the satellite group on day 56. These values were all within the normal range, and there was no significant change in K^+^ in the high- and low-dose groups, suggesting that subtilisin QK had non-adverse effects on the concentration of K^+^ in rat serum. TBIL is an important indicator of jaundice, and the liver plays an important role in the metabolism of bilirubin [27]. Compared with the control group, TBIL in 7500 FU/kg males was increased on day 28 (*p* < 0.05). However, it was also within the normal range, and there was no significant change in TBIL in the high- and low-dose groups and satellite groups. The urea results showed that there was a statistically significant decrease in the 2500, 7500, and 25,000 FU/kg male satellite groups on day 56 compared to the control group. However, the values were are all within the normal range, and there were no dose-related differences. In addition, there were no significant differences in the dosing groups on day 28, or in the female satellite groups on day 56. Hence, the changes were considered unlikely to be treatment-related with no toxicological significance. In addition, there was no significant abnormalities in the body weights, food consumption, organ weights, and macroscopic findings. Pathological examination also did not show treatment-related changes. The results were similar to those obtained after repeated dosing of nattokinase in rats and mice in previous studies [19, 28]. These results showed that the maximum dose of subtilisin QK had no significant toxic effects in this study.

Because no significant adverse effects were observed in the acute and subchronic toxicity studies, we further conducted a safety pharmacological study in SD rats, which was used to determine the predicted and unpredicted side effects of the drugs [29]. In the safety pharmacology assessment, the effects of a single administration of subtilisin QK on the function of the central nervous system in rats was observed. The results showed that subtilisin QK did not cause adverse effects on the central nervous system. The highest doses in the acute toxicity and 28-day subchronic toxicity studies were 100,000 FU/kg and 25,000 FU/kg, which were approximately 600 and 150 times more than the recommended daily dose for humans, respectively. At such high doses, the subtilisin QK dose group did not have a significant effect on the physiological state of the rats, nor did it produce significant toxicity to tissues and organs. These results suggest that subtilisin QK will be safe when applied at a normal dose in humans.

## Conclusion

In conclusion, this study evaluated the acute toxicity, 28-day subchronic toxicity, and safety pharmacology of subtilisin QK oral in SD rats. In the acute toxicity study, a single oral dose of 100,000 FU/kg subtilisin QK caused no adverse effects. In the subchronic toxicity study, no obvious clinical symptoms or evidence of organ-specific toxicity in SD rats at a daily dose up to 25,000 FU/kg subtilisin QK were observed. In the safety pharmacology study, no adverse effects on the central nervous of SD rats post-administration of up to of 5000 FU/kg subtilisin QK occurred. The results indicated that the oral consumption of subtilisin QK was very safe for SD rats, with a no-observed-adverse-effect level of 25,000 FU/kg.

## Supplementary Information

**Additional file 1.** The online version of this article contains supplementary materials.

## Data Availability

The datasets generated during and/or analyzed during the current study are available from the corresponding author on reasonable request.
