# Supplementary material for: Safety assessment of subtilisin QK in rats
Source: BMC Pharmacol Toxicol. 2021 Jun 26;22:38. doi: 10.1186/s40360-021-00506-w (PMC8235616; doi:10.1186/s40360-021-00506-w)
Supplement: Supplementary file 1 — Additional file 1. The online version of this article contains supplementary materials. [file 40360_2021_506_MOESM1_ESM.docx]

**Supplementary date**

**Supplementary table 1** Subtilisin QK specification and data from three batches

**Supplementary table 2** Body weight and food consumption of Sprague Dawley rats in the acute toxicity study of Subtilisin QK

**Supplementary table 3** Hematological parameters of Sprague Dawley rats administration with subtilisin QK for 28 days and 28days recovery

**Supplementary Table 4** Functional observation battery (FOB) and function tests parameters of Sprague Dawley rats administration with subtilisin QK for Safety pharmacology

**Supplementary Fig.1** Representative H&E stains of organs from male and female rats was exposed to 0 FU/kg (Control) and 25000 FU/kg of Subtilisin QK

**Supplementary Table 1** Subtilisin QK specification and data from three batches

|  | specification | batch 1 | batch 2 | batch 3 |
| --- | --- | --- | --- | --- |
| Weight | - | 2.30 kg | 2.35 kg | 2.25 kg |
| Identity | confirms | confirms | confirms | confirms |
| Character | Light yellow powder | confirms | confirms | confirms |
| Subtilisin QK Activity | NLT 20000 FU/g | 27500 FU/g | 25600 FU/g | 27900 FU/g |
| Molecular weight | 27.7±2.7kDa | 27.1 kDa | 27.0 kDa | 26.7 kDa |
| Protein percentage | ≥10mg/g | 15.54 mg/g | 14.75 mg/g | 15.98 mg/g |
| Protein content | ≥35% | 37.64% | 37.40% | 36.17% |
| moisture | ≤ 9.0% | 7.70% | 7.61% | 7.78% |
| PH | 5~8 | 7.09 | 7.22 | 6.94 |
| Total viable aerobic count | NMT 100 CFU/g | NMT 100 CFU/g | NMT 100 CFU/g | NMT 100 CFU/g |
| Yeast/Mold | NMT 100 CFU/g | NMT 10 CFU/g | NMT 10 CFU/g | NMT 10 CFU/g |
| E. coli | Not detected in 25 g | Negative | Negative | Negative |

NLT, Not less than; NMT, Not more than.

**Supplementary table 2** Body weight and food consumption of Sprague Dawley rats in the acute toxicity study of Subtilisin QK

|  | | Male | |  | Female | |
| --- | --- | --- | --- | --- | --- | --- |
|  | | Control | 100000FU/kg |  | Control | 100000FU/kg |
| Body weight（g）  n=10 | D0 | 187.1±8.7 | 187.7±9.5 |  | 169.0±7.3 | 171.5±8.2 |
|  | D2 | 211.5±9.3 | 209.2±10.1 |  | 184.9±8.1 | 188.0±12.4 |
|  | D7 | 240.9±8.8 | 236.2±14.3 |  | 202.9±8.4 | 204.4±11.1 |
|  | D14 | 288.6±10.6 | 288.1±19.4 |  | 218.5±11.1 | 220.9±11.6 |
| Food consumption (g)  n=10 | D2 | 24.75 | 23.3 |  | 20.75 | 19.75 |
|  | D7 | 30.2 | 30.3 |  | 20 | 20.4 |
|  | D14 | 35.25 | 33.05 |  | 18.75 | 21.15 |

**Supplementary Table 3** Hematological parameters of Sprague Dawley rats administration with subtilisin QK for 28 days and 28days recovery

|  |  |  | male | | | |  | female | | | |
| --- | --- | --- | --- | --- | --- | --- | --- | --- | --- | --- | --- |
|  |  | Normal range | Control | 2500 FU/kg | 7500 FU/kg | 25000 FU/kg |  | Control | 2500 FU/kg | 7500 FU/kg | 25000 FU/kg |
| D_28_ | WBC (10^9^/L) | 3.5-9.7 | 4.95±2.43 | 4.63±1.80 | 4.95±2.74 | 4.29±1.94 |  | 4.10±1.65 | 4.26±0.97 | 3.52±1.61 | 3.74±1.61 |
| n=15 | NEUT# (10^9^/L) | 0-1.2 | 0.66±0.42 | 0.60±0.24 | 0.73±0.32 | 0.68±0.21 |  | 0.52±0.34 | 0.55±0.24 | 0.37±0.18 | 0.46±0.30 |
|  | LYMPH# (10^9^/L) | 0.34-11.04 | 4.23±2.08 | 3.96±1.62 | 4.14±2.39 | 3.55±1.84 |  | 3.53±1.42 | 3.64±0.84 | 3.10±1.53 | 3.23±1.51 |
|  | MONO# (10^9^/L) | 0-0.54 | 0.02±0.02 | 0.04±0.03 | 0.04±0.05 | 0.03±0.02 |  | 0.03±0.02 | 0.03±0.03 | 0.02±0.02 | 0.03±0.02 |
|  | EO# (10^9^/L) | 0-1.0 | 0.04±0.02 | 0.03±0.02 | 0.04±0.02 | 0.04±0.02 |  | 0.03±0.01 | 0.04±0.01 | 0.03±0.01 | 0.02±0.01 |
|  | BASO# (10^9^/L) | 0-0.03 | 0.00±0.00 | 0.00±0.00 | 0.00±0.00 | 0.00±0.00 |  | 0.00±0.00 | 0.00±0.00 | 0.00±0.00 | 0.00±0.00 |
|  | NEUT% (%) | 9-34 | 13.26±4.04 | 13.50±4.83 | 15.46±2.52 | 17.37±6.15 |  | 12.35±5.04 | 12.73±4.84 | 11.12±4.99 | 13.05±6.92 |
|  | LYMPH% (%) | 65-85 | 85.35±4.28 | 85.03±5.10 | 82.96±2.88 | 80.96±6.53 |  | 86.09±5.35 | 85.68±5.19 | 87.52±4.91 | 85.53±7.12 |
|  | MONO% (%) | 0-5 | 0.48±0.38 | 0.70±0.49 | 0.79±0.49 | 0.62±0.40 |  | 0.83±0.48 | 0.72±0.49 | 0.59±0.50 | 0.74±0.40 |
|  | EO% (%) | 0-6 | 0.91±0.26 | 0.77±0.41 | 0.79±0.41 | 1.05±0.82 |  | 0.73±0.44 | 0.87±0.33 | 0.77±0.31 | 0.68±0.34 |
|  | BASO% (%) | 0-1.5 | 0.00±0.00 | 0.00±0.00 | 0.00±0.00 | 0.00±0.00 |  | 0.00±0.00 | 0.00±0.00 | 0.00±0.00 | 0.00±0.00 |
|  | RBC (10^12^/L) | 5.45-8.12 | 7.50±0.40 | 7.33±0.36 | 7.16±0.38 | 7.63±0.33 |  | 7.60±0.35 | 7.44±0.43 | 7.69±0.23 | 7.35±0.22 |
|  | HGB(g/L) | 118.47-156.97 | 147.40±6.59 | 145.90±8.46 | 145.90±6.62 | 148.60±4.53 |  | 147.10±4.65 | 144.30±4.85 | 146.40±3.63 | 142.00±4.78 |
|  | HCT (%) | 34.3-51.9 | 44.01±2.02 | 42.79±1.93 | 43.54±2.01 | 45.46±1.44 |  | 43.37±1.62 | 43.07±1.56 | 43.74±1.42 | 41.66±1.61 |
|  | MCV (fL) | 80-101 | 58.76±2.19 | 58.45±1.98 | 60.84±1.81 | 59.62±2.29 |  | 57.08±2.07 | 57.99±1.64 | 56.92±1.52 | 56.70±1.04 |
|  | MCH (pg) | 26.4-34.7 | 19.66±0.36 | 19.91±0.68 | **20.38±0.57*** | 19.49±0.63 |  | 19.37±0.62 | 19.43±0.58 | 19.04±0.47 | 19.34±0.36 |
|  | MCHC (g/L) | 313-361 | 335.20±6.78 | 340.80±5.55 | 335.20±5.45 | **327.10±7.03*** |  | 339.20±4.57 | 335.20±2.35 | 334.80±5.39 | 341.00±4.06 |
|  | PLT (10^9^/L) | 500-1300 | 1213.60±117.74 | 1197.20±121.53 | 1135.40±126.06 | 1110.70±133.79 |  | 1104.40±133.51 | 1140.30±164.61 | 1100.50±116.06 | 1050.10±125.36 |
|  | RET# (10^9^/L) | 140.7-595.4 | 291.35±41.53 | 280.69±36.40 | 307.94±39.96 | 290.40±26.95 |  | 241.02±41.72 | 224.71±28.72 | 242.56±42.89 | 231.16±45.76 |
| D_56_ | WBC (10^9^/L) | 3.5-9.7 | 6.98±2.73 | 6.24±1.66 | 6.45±0.95 | 7.44±2.87 |  | 3.65±0.27 | 4.31±1.78 | 4.14±0.99 | 3.01±0.83 |
| n=5 | NEUT# (10^9^/L) | 0-1.2 | 0.93±0.20 | 0.80±0.28 | 0.76±0.21 | 1.30±0.65 |  | 0.60±0.08 | 0.76±0.43 | 0.57±0.17 | 0.49±0.10 |
|  | LYMPH# (10^9^/L) | 0.34-11.04 | 5.94±2.74 | 5.31±1.54 | 5.58±0.77 | 5.99±2.29 |  | 2.99±0.32 | 3.45±1.42 | 3.48±0.89 | 2.45±0.84 |
|  | MONO# (10^9^/L) | 0-0.54 | 0.05±0.04 | 0.04±0.03 | 0.03±0.02 | 0.08±0.05 |  | 0.03±0.01 | 0.04±0.02 | 0.03±0.02 | 0.02±0.01 |
|  | EO# (10^9^/L) | 0-1.0 | 0.06±0.01 | 0.09±0.08 | 0.08±0.05 | 0.07±0.04 |  | 0.03±0.01 | 0.06±0.03 | 0.06±0.04 | 0.04±0.02 |
|  | BASO# (10^9^/L) | 0-0.03 | 0.00±0.00 | 0.00±0.00 | 0.00±0.00 | 0.00±0.00 |  | 0.00±0.00 | 0.00±0.00 | 0.00±0.00 | 0.00±0.00 |
|  | NEUT% (%) | 9-34 | 14.52±4.99 | 13.30±4.70 | 11.74±2.31 | 17.28±4.06 |  | 16.48±3.04 | 17.10±4.25 | 13.94±3.12 | 17.12±5.54 |
|  | LYMPH% (%) | 65-85 | 83.80±4.95 | 84.74±5.15 | 86.62±2.73 | 80.64±4.06 |  | 81.78±3.11 | 79.88±4.33 | 83.82±4.01 | 80.42±6.02 |
|  | MONO% (%) | 0-5 | 0.68±0.46 | 0.72±0.52 | 0.50±0.29 | 1.14±0.50 |  | 0.80±0.27 | 1.06±0.72 | 0.76±0.40 | 0.86±0.40 |
|  | EO% (%) | 0-6 | 1.00±0.40 | 1.24±0.77 | 1.14±0.56 | 0.94±0.24 |  | 0.94±0.30 | 1.96±2.27 | 1.48±0.82 | 1.60±0.77 |
|  | BASO% (%) | 0-1.5 | 0.00±0.00 | 0.00±0.00 | 0.00±0.00 | 0.00±0.00 |  | 0.00±0.00 | 0.00±0.00 | 0.00±0.00 | 0.00±0.00 |
|  | RBC (10^12^/L) | 5.45-8.12 | 7.96±0.34 | 8.02±0.36 | 7.93±0.37 | 7.94±0.42 |  | 7.45±0.31 | 7.44±0.37 | 7.44±0.25 | 7.51±0.16 |
|  | HGB(g/L) | 118.47-156.97 | 146.80±5.93 | 147.40±7.50 | 145.40±5.27 | 147.80±7.98 |  | 143.00±7.04 | 140.40±4.10 | 140.80±2.49 | 141.60±3.36 |
|  | HCT (%) | 34.3-51.9 | 42.84±1.40 | 43.22±1.70 | 43.02±1.40 | 43.48±2.25 |  | 41.80±1.89 | 40.76±1.19 | 40.68±0.28 | 41.28±0.73 |
|  | MCV (fL) | 80-101 | 53.82±1.69 | 53.92±0.86 | 54.28±0.88 | 54.78±1.59 |  | 56.08±0.63 | 54.86±1.61 | 54.78±2.07 | 54.96±1.57 |
|  | MCH (pg) | 26.4-34.7 | 18.44±0.69 | 18.40±0.29 | 18.36±0.53 | 18.62±0.41 |  | 19.20±0.21 | 18.88±0.54 | 18.94±0.76 | 18.88±0.52 |
|  | MCHC (g/L) | 313-361 | 342.40±4.93 | 341.00±5.15 | 338.20±5.26 | 339.80±2.68 |  | 342.00±5.96 | 344.60±4.04 | 346.00±5.43 | 343.00±2.92 |
|  | PLT (10^9^/L) | 500-1300 | 1092.40±155.73 | 1137.40±89.03 | 1081.20±96.36 | 1038.40±115.19 |  | 1025.40±155.37 | 935.40±235.21 | 1124.80±108.15 | 1037.00±145.02 |
|  | RET# (10^9^/L) | 140.7-595.4 | 262.10±25.60 | 215.30±40.64 | 278.66±35.56 | 261.28±51.42 |  | 174.40±38.53 | 183.92±36.51 | 189.52±16.36 | 194.16±37.97 |

**p* <0.05.

WBC, White blood cell; RBC, Red blood cell; HGB, Hemoglobin; HCT, Hematocrit; MCV, Mean corpuscular volume; MCH, Mean corpuscular hemoglobin; MCHC, Mean corpuscular hemoglobin concentration; PLT, Platelet; RET %, RET #, Reticulocyte count; NEUT #, Neutrophil count; LYMPH #, Lymphocyte count; MONO #, Monocyte count; EO #, Eosinophil count; BASO #, Basophils count; NEUT %, Neutrophil ratio; LYMPH %, Lymphocyte ratio; MONO %, Monocyte ratio; EO %, Eosinophil ratio; BASO %, Basophils ratio.

**Supplementary Table 4** Functional observation battery (FOB) and function tests parameters of Sprague Dawley rats administration with subtilisin QK for Safety pharmacology

|  |  | prior | 0.5h | 2h | 4h | 6h | 8h | 24h |
| --- | --- | --- | --- | --- | --- | --- | --- | --- |
| Body temperature (℃) | Control | 37.5±0.3 | 38.2±0.4 | 37.9±0.6 | 37.2±0.3 | 37.3±0.2 | 37.2±0.5 | 37.0±0.3 |
| n=10 | 500 FU/kg | 37.4±0.4 | 38.1±0.4 | 37.8±0.5 | 37.3±0.3 | 37.1±0.2 | 37.2±0.3 | 37.1±0.5 |
|  | 1500 FU/kg | 37.5±0.5 | 38.2±0.4 | 37.9±0.6 | 37.5±0.4 | 37.4±0.4 | 37.5±0.4 | 37.3±0.4 |
|  | 5000 FU/kg | 37.6±0.6 | 38.3±0.5 | 38.0±0.3 | 37.6±0.3 | 37.7±0.4 | 37.5±0.4 | 37.5±0.4 |
| Total active distance (mm) | Control | 6359±2417 | 3462±2315 | 4880±2448 | 2911±1987 | 2183±1648 | 3196±3016 | 4425±2153 |
| n=10 | 500 FU/kg | 5734±3043 | 2242±2580 | 2416±1958 | 2161±2620 | 1086±1444 | 1370±1775 | 3256±2342 |
|  | 1500 FU/kg | 6432±2250 | 3352±3018 | 3626±1956 | 2903±2825 | 2331±1976 | 2640±2267 | 4178±1897 |
|  | 5000 FU/kg | 6460±2479 | 2520±2004 | 3283±2393 | 1420±1486 | 617±732 | 1506±1600 | 4015±1964 |
| Number of activities | Control | 27±6 | 34±9 | 28±10 | 27±6 | 27±11 | 26±13 | 31±9 |
| n=10 | 500 FU/kg | 22±8 | 21±18 | 26±15 | 20±16 | 18±13 | 16±15 | 23±12 |
|  | 1500 FU/kg | 29±8 | 24±11 | 30±8 | 22±9 | 23±7 | 27±6 | 32±7 |
|  | 5000 FU/kg | 28±6 | 25±10 | 26±8 | 21±9 | 17±10 | 19±10 | 29±7 |
| Holding power(g) | Control | 910±147 | 941±108 | 873±105 | 879±141 | 855±59 | 890±117 | 925±143 |
| n=10 | 500 FU/kg | 865±155 | 935±134 | 851±148 | 892±111 | 783±109 | 803±132 | 879±102 |
|  | 1500 FU/kg | 876±134 | 909±109 | 871±107 | 835±154 | 809±102 | 833±94 | 847±162 |
|  | 5000 FU/kg | 898±72 | 871±155 | 796±106 | 795±86 | 742±91 | 804±94 | 856±109 |


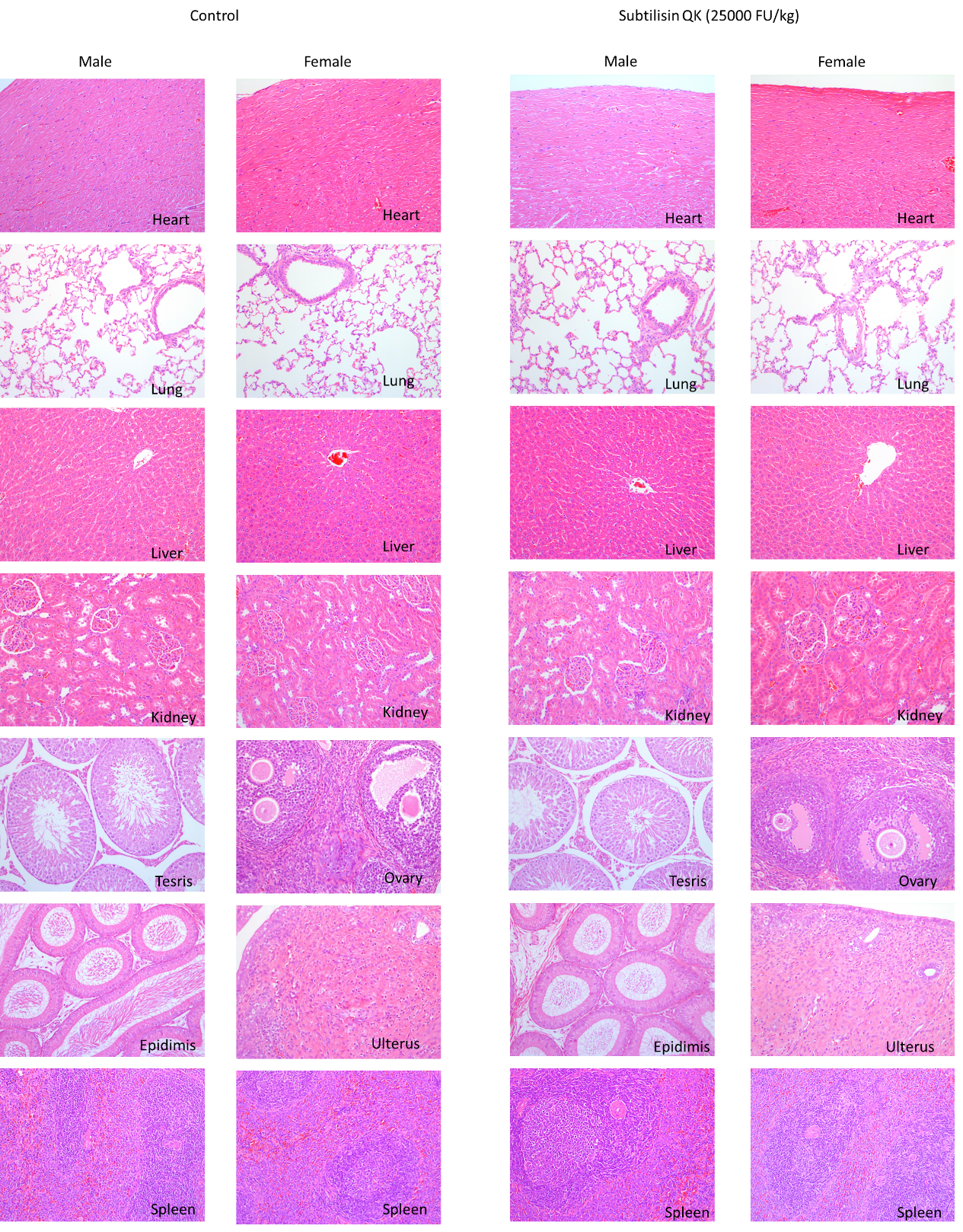


**Supplementary Fig.1** Representative H&E stains of organs from male and female rats was exposed to 0 FU/kg (Control) and 25000 FU/kg of Subtilisin QK. The photographs imaged at 200╳.
